# Designing an Inclusive Learning Training Series for Pharmacy Educators

**DOI:** 10.3390/pharmacy10050113

**Published:** 2022-09-13

**Authors:** Jacqueline E. McLaughlin, Kathryn A. Morbitzer, Bethany Volkmar, Suzanne C. Harris, Charlene R. Williams, Michael D. Wolcott, Michael B. Jarstfer, Carla Y. White

**Affiliations:** 1 UNC Eshelman School of Pharmacy, University of North Carolina, Chapel Hill, NC 27599, USA; 2 Workman School of Dental Medicine, High Point University, High Point, NC 27268, USA

**Keywords:** inclusive teaching, diversity, evaluation, faculty development

## Abstract

This article describes the design, implementation, and evaluation of five faculty development sessions focused on inclusive teaching strategies in pharmacy education. Inclusive strategies ensure that every student can clearly understand and engage in meaningful learning opportunities. Three sessions were implemented in fall 2020 and two in spring 2021. Sessions focused on experiential, didactic, and graduate education. A convergent parallel mixed methods evaluation was conducted using descriptive statistics and thematic analysis. Sessions were highly rated, and participants provided suggestions for curriculum improvement (e.g., creating resources, surveying students, and peer auditing syllabi for aspects of inclusiveness). Given the increasing emphasis on inclusion in pharmacy education, this work is timely for sharing strategies aimed at faculty development and teaching practices.

## 1. Introduction

Teaching practices that effectively promote student development are critical to preparing the next generation of healthcare providers [1]. Studies indicate that, although health professions educators are experts in the content they teach, they rarely receive training on effective teaching practices [2,3]. While most educator training focuses on how we teach, a contemporary emphasis on who we teach is emerging [4,5,6]. 

This shift highlights the importance of inclusive teaching strategies, which enable all students to clearly understand and engage in meaningful learning opportunities [6,7]. Inclusive activities and environments ensure that every student can participate, pose ideas, construct knowledge, see personal connections to the topic, and feel welcomed into intellectual discussions [6]. Without attention to the structure of classroom interactions, content and learning may only be accessed by a subset of students and can lead to feelings of exclusion, unfairness, and discordance between cultural backgrounds and the learning environment [6]. 

To that end, pharmacy educators must develop the pedagogical skills needed to meet our increasingly diverse environments [5]. Pharmacy schools embody a wide range of learners and learning environments, including Bachelor’s, Master’s (MS), Doctor of Pharmacy (PharmD), and Doctor of Philosophy (PhD) students learning within the classroom, laboratory, and experiential settings. In addition, many schools are actively working to increase compositional diversity with the goal of preparing learners to serve increasingly diverse patients [8,9,10,11,12,13,14,15]. However, research suggests that most educators lack the necessary training to address these issues [16]. 

In 2020–2021, the UNC Eshelman School of Pharmacy’s Center for Innovative Pharmacy Education and Research (CIPhER) partnered with the School’s Office of Organizational Diversity and Inclusion (ODI) and Office of Experiential Programs (OEP) to offer 5 educator development sessions focused on inclusive learning. They were aligned with CIPhER core aims (i.e., educator development), the ODI Strategic Plan (i.e., Strategic Priority III: Build an Inclusive Community), and the school’s Beyond Strategic Plan (i.e., Priority 1, Objective 1: Foster Diversity, Equity, & Inclusion (DEI)) [17]. The purpose of this article is to describe the design, implementation, and evaluation of the sessions.

## 2. Materials and Methods

As a starting point for the development of the inclusive learning series, the CIPhER team and Associate Dean for ODI conceptualized the overarching goals of the sessions:

1. Recognize examples of bias, stereotypes, discrimination, and microaggressions, both in themselves and others;
2. Explain how inclusive learning practices positively impact student learning and how the lack of inclusivity negatively impacts the student experience; and
3. Apply strategies on how to implement, facilitate, and communicate inclusive pedagogical practices in teaching, precepting, and mentoring.

Additionally, it was determined that different strategies and examples may be unique to different learning topics and contexts. Therefore, the learning series included five sessions focused on various educational settings (e.g., precepting and classroom teaching). 

Advertisements and registration information were sent to all school faculty, staff, postdoctoral trainees, preceptors and students, as well as any person outside UNC who participated in a previous CIPhER event. Presenters had experience related to the session topic. Sessions geared towards participants associated with the didactic curriculum took place during our school’s lunch hour when no classes were held. Sessions geared towards participants associated with the experiential curriculum took place in the afternoon at a time when most patient care activities would have been completed for the day. All sessions were offered online via Zoom; they were also recorded and made available to individuals by request. Three sessions were held during fall 2020, and two sessions were held in spring 2021 (Appendix A, Table A1).

Session 1. Creating an Inclusive Learning Environment in the Residential Setting

This program targeted instructors in the didactic curriculum. The session included definitions related to inclusive learning and evidence of the impact of inclusive learning approaches on student learning. Strategies were provided on inclusive student engagement and inclusive course design. Small group breakout discussions and large group debriefs were used to provide real-life examples related to student engagement and pharmacotherapy case writing. Considerations for syllabus language were discussed.

Session 2. Creating a Racially Inclusive Learning Environment in the Experiential Setting

This program targeted experiential educators and started with definitions related to inclusive learning. The presenters introduced the three zones of action: head (urges one to think outside their personal experience); heart (emphasizes feelings and empathy); and hands (urges one to take action in advocating and modifying biases/practices that undermine diversity). Participants were asked to reflect on a time they felt uncomfortable discussing race in the learning environment. Real-life scenarios were used as examples to stimulate discussion (e.g., preceptor hearing an elderly patient state a derogatory remark to a Black student). Presenters shared strategies for inclusive learning, such as the AAA framework to improve communication (acknowledge, ask, and adapt) [18].

Session 3. Creating an Inclusive Learning Environment in the Graduate Education Setting

This session targeted educators associated with the graduate program (i.e., PhD and MS). The presenters provided definitions and graduate education examples for terms such as microaggressions, implicit bias, stereotyping, and privilege. The unique role that graduate education faculty can play in creating an inclusive learning environment as instructors and research advisors was discussed. Scenarios provided for discussion included examples related to inclusivity in the lab environment and the admissions process.

Session 4. Considerations of the LGBTQIA+ Community in Creating an Inclusive Learning Environment

This session targeted educators in the experiential curriculum and/or didactic curriculum. The presenters started with an overview of different gender identities and the scope of the LGBTQIA+ community. Small group breakout and large group discussion focused on one example of a student who was transitioning gender and another example of a colleague making a derogatory remark about a learner who identifies as gay. Participants were provided frameworks and strategies to help them reflect on their own teaching practices with an emphasis on responding to inappropriate language use.

Session 5. inclusifiED: Inclusive Teaching Practices Workshop

The last session was designed as a workshop for participants associated with the didactic curriculum. The presenters asked participants to reflect on inequities and diversity in their classrooms through interactive activities. After providing a framework for inclusive design and their own research results, the presenters led participants through active learning exercises and case studies that explored inclusive techniques and modeled approaches. 

Convergent parallel mixed methods were used to evaluate the learning series. Post-event surveys were administered anonymously online following each session. No incentives were provided for survey completion. Three items related to the value of what was learned, the quality of the session, and the effectiveness of the presenter were measured on a 5-point scale from 1—very low to 5—very high. Four open-text items queried aspects of the session that were most useful, aspects that could have been improved, questions that remained unanswered, and ways the topic could be used to improve courses/curricula. Quantitative items were analyzed using descriptive statistics; given the sample size and approximately normal distribution of the data, mean and standard deviation were used [19]. Qualitative items were analyzed using thematic analysis by two researchers, who reviewed and identified themes independently and worked together to reach a consensus as needed [20]. Summary reports were created for each session, shared with the presenter(s) for feedback, and provided to school leadership. This evaluation was submitted to the UNC IRB and determined to be exempt from full review. 

## 3. Results

There were 273 attendances across all 5 sessions and 142 completed post-program evaluations (response rates 32.4–68.2%). Attendees included school faculty (*n* = 102 (37.5%)), preceptors (*n* = 119 (43.6%)), postdoctoral trainees (*n* = 16 (5.7%)), students (*n* = 8 (3.0%)), and staff (*n* = 28 (10.2%)). Most attendees were affiliated with the school (*n* = 232 (85.0%)). Nearly all school faculty attended at least one session. Fifty-five participants (20.1%) participated in more than one session. Of those 55 participants, 33 participated in session 5.

Table 1 provides details regarding attendance and evaluation results. Overall, 95.7% of participants rated the overall value of what they learned as very high or high, 97.9% rated the overall quality of the session as very high or high, and 95.8% rated the overall effectiveness of the presenter as very high or high. 

Thematic findings of open-text comments supported survey findings regarding value, quality, and presenter effectiveness. When asked about aspects that were most useful, participants most frequently noted the case studies, group discussions, content, expert speakers, and honest dialogue. For example, one participant noted, “I think the presenters did a great job of setting the tone and creating a safe space to have these conversations”, while another stated, “Thanks to the presenters for being honest and open and doing the labor to help (us) be inclusive to our students!”—providing depth and alignment with the high marks seen in the surveys for presenter effectiveness. Similarly, comments provided details regarding what was valued, such as, “Sharing that it is okay for us as preceptors to make mistakes, knowing the importance of apologizing when we do” and “I liked having the breakout room; the cases were great and allowed for an environment to have conversations that are typically avoided.”.

When asked about aspects to improve, participants most frequently requested longer seminars, more time in group discussion, additional instructions for group discussion, making the sessions mandatory for faculty, including additional learner perspectives or examples, and offering more DEI programming. After the programs, participants still had questions about how to promote inclusion within their programs, how to attend more programming, and who to contact for more support and training. When asked about ways the topic could be used to improve courses and curricula, participants suggested that the training be required, used to improve learning environments, applied to addressing difficult situations, and integrated into patient cases (Table 2). 

## 4. Discussion

Inclusive teaching focuses on creating learning environments that enable students to effectively construct knowledge by leveraging student experience, attitude, motivation, confidence, and participation [6,21,22]. The findings of our evaluation align with other studies that indicate a desire for more inclusive training and illustrate positive participant perceptions associated with culturally related training [8,23,24,25,26,27,28,29]. Preceptors in our study, for example, indicated that they desired more inclusive training, which aligns with the results of a previous preceptor needs assessment [29]. Furthermore, creating an atmosphere of emotional well-being and mutual respect results in students feeling comfortable asking questions, expressing their thoughts and feelings, fostering safety, promoting collaboration, and building a sense of community [30,31,32,33]. 

A unique aspect of this work was the scope of the training, which included the teaching of graduate students. There is a unique need for inclusivity training for PhD educators, specifically, since a large portion of PhD training is conducted with a single primary research advisor and associated laboratory. Ongoing challenges in PhD training related to DEI underscore the importance of this work [34,35,36]. 

While the training series described in this paper focused on inclusive learning, other frameworks might be useful for this purpose. For example, culturally responsive teaching incorporates students’ cultural knowledge, prior experiences, and frames of reference to add relevance and culture to content [5,37]. Related, culturally relevant pedagogy focuses on the importance of culture in schooling and requires teachers to be inclusive of student backgrounds in order to be effective [38]. 

Providing educator development can help increase motivation and enthusiasm for teaching, improve knowledge and behaviors, and promote skill development [39]. However, this evaluation is limited to participants’ perception of value, quality and teacher effectiveness (i.e., level 1 of Kirkpatrick’s pyramid). While this is an important step, some research suggests that additional strategies and follow-up are needed beyond workshops or seminars to improve the uptake of educator development, such as mentoring, coaching, and feedback [38]. More work is needed to better understand the potential impact of the training on critical outcomes, such as learning effectiveness, intention to change teaching strategies, and changes in teaching practices. 

That being said, numerous pharmacy organizations continue to emphasize the need for inclusivity [40,41,42,43]. The American Society of Health System Pharmacists, for example, states that a diverse and culturally competent healthcare workforce is essential in reducing racial and ethnic healthcare disparities [40,41]. Similarly, the American Pharmaceutical Association established its Health Equity Committee to achieve social justice goals by monitoring the diversity of its sections, committees, councils, and boards [43]. Recognizing systemic challenges that perpetuate inequality in healthcare can help pharmacy schools develop organizational strategies that support the development of an inclusive and culturally intelligent workforce [4]. 

There are several limitations to this work worth noting. First, participation in the training was voluntary, which may have introduced self-selection bias. Second, the trainings were offered by a single institution, which limits generalizability. Third, the evaluation focused on perceptions, leaving the effectiveness of the training unclear. Fourth, the trainings focused on topics considered a priority for the institution and may not have included other important areas (e.g., English language skills). Fifth, the sessions were offered online only, which could have impacted access and engagement. Despite these limitations, this work provides (a) an approach to training educators for inclusive learning that may be useful across pharmacy education and (b) participant suggestions for incorporating inclusive teaching strategies within various pharmacy education contexts. This work advances a small yet growing body of literature exploring diversity, bias, and inclusive training within pharmacy education [4,44]. More work is needed to explore and understand how to optimize and evaluate inclusive training. 

## 5. Conclusions

Pharmacy educators should take steps to cultivate equitable classroom environments that promote engagement and learning. This paper described the design and implementation of an inclusive teaching series, along with curricular opportunities, that might be useful for other schools considering this topic for faculty and preceptor development. Given the increasing emphasis on DEI in pharmacy education, this work is timely for sharing strategies aimed at faculty development and teaching practices.

## Figures and Tables

**Table 1:** Details and results of post-program evaluations.

Session	Participants	Value *Mean* (*SD*)	Quality *Mean* (*SD*)	Presenter Effectiveness *Mean* (*SD*)
1. Residential/Classroom	N = 43 (*n* = 22)	4.5 (0.6)	4.7 (0.5)	4.6 (0.7)
2. Precepting/Clinical	N = 86 (*n* = 46)	4.7 (0.5)	4.8 (0.5)	4.8 (0.5)
3. Graduate	N = 37 (*n* = 12)	4.4 (0.8)	4.4 (0.8)	4.4 (0.8)
4. LGBTQIA+	N = 44 (*n* = 30)	4.8 (0.4)	4.8 (0.4)	4.8 (0.5)
5. inclusifiED	N = 63 (*n* = 32)	4.8 (0.6)	4.9 (0.3)	4.8 (0.4)
***Total***	N = 273 (*n* = 142)	4.7 (0.5)	4.8 (0.5)	4.7 (0.5)

N = number of attendees; *n* = number of evaluation responses; SD = standard deviation; items measured on a scale from 1 (very low) to 5 (very high).

**Table 2:** Themes from participant responses to the survey question, “In what ways do you think this topic could be used to improve our courses/curricula or educational programs?”.

Session	Participant Ideas for Applying the Topic
**1.** **Residential/Classroom**	Case development; in-class interactions; create and train student groups to be more inclusive and diverse; fuse empathy for students into the design of the course, assignment, and activities
**2.** **Precepting/Clinical**	Incorporate into every training level (e.g., PharmD, post-graduate training, precepting); case development; include as a learning module for hospital employees; incorporate into each course; create a toolkit in learning management system with these resources
**3.** **Graduate**	Each division could have a seminar dedicated to diversity topics, which could be a course for Ph.D. students; have DEI conversations about the graduate program and how we can improve; increase awareness of DEI issues
**4.** **LGBTQIA+**	Case development; training on how to communicate appropriately and respectfully; more sessions regarding this topic; support learners from this community; incorporate into all preparatory materials for educators, preceptors, etc.
**5.** **inclusifiED**	Design courses to leverage differences in students and promote student engagement; survey the class to discover factors that may affect student learning; peer audit faculty syllabi for inclusiveness; pace content delivery and gauge student comfort with material

## Data Availability

Data available upon request.

## Appendix A

Summary of Inclusive Learning Series Sessions

**Table A1:** Details of each Session.

Session Title	Session Objectives	Presenters	Duration	Session Activities	Example Topics Covered
Creating an Inclusive Learning Environment in the Residential Setting	Recognize examples of unconscious bias, stereotype threat, privilege, and microaggressions, both in themselves and in others Explain how inclusive classroom practices positively impact student learning and how the lack of inclusivity or the presence of unconscious bias might negatively impact the student experience Obtain strategies to implement and facilitate inclusive pedagogical practices in teaching and course design	1	60 min	Imagery exercise and reflection Small group zoom breakout discussion Large group discussion	Definitions related to inclusive learning (e.g., microaggressions, implicit bias, stereotype threat, unintentional privilege) Evidence demonstrating the impact of inclusive learning approaches on student learning Unconscious bias in student engagement Considerations for inclusive course design (e.g., case writing/discussions, inclusive syllabus, student discourse)
Creating a Racially Inclusive Learning Environment in the Experiential Setting	Define key terms to enhance racially inclusive learning practices Explain how inclusive experiential practices positively impact learning and how the lack of inclusivity or the presence of unconscious bias might negatively impact the learner experience Describe strategies to have racially sensitive conversations and responses	4	60 min	Reflection activity Small group zoom breakout discussion Large group discussion	Three Zones of Action framework (i.e., head, heart, hands) AAA Framework (i.e., acknowledge, ask, adapt) Considerations for creating an inclusive learning environment (e.g., helping students deal with derogatory comments from patients) Workplace resources
Creating an Inclusive Learning Environment in the Graduate Education Setting	Recognize examples of unconscious bias, stereotype threat, privilege, and microaggressions, both in themselves and in others Explain how inclusive practices in the laboratory, classroom, and other group settings positively impact student learning and how the lack of inclusivity or the presence of unconscious bias might negatively impact the student experience Recognize the perspectives and experiences of graduate students in relation to feelings of diversity and inclusion List strategies to implement and facilitate inclusive pedagogical practices in research mentoring, teaching, and advising	2	60 min	Small group zoom breakout discussion Large group discussion	Definitions related to inclusive learning (e.g., microaggressions, implicit bias, stereotype threat, unintentional privilege) Evidence of the need for diversity in STEM (incl. DEI anecdotes from school) Overview of school’s DEI Strategic Plan in relation to graduate program Considerations for roles that graduate program faculty can play in creating an inclusive learning environment (e.g., in the classroom, lab, admissions process)
Considerations of the LGBTQIA+ Community in Creating an Inclusive Learning Environment	Define key terms to enhance inclusive learning practices Explain how inclusive educational (or teaching) practices positively impact learning and how the lack of inclusivity or the presence of unconscious bias might negatively impact the learner experience Describe strategies to have sensitive conversations and responses	3	60 min	Small group zoom breakout discussion Large group discussion	Overview of different gender identities and the scope of the LGBTQIA+ community AAA Framework (i.e., acknowledge, ask, adapt) LIFT Framework (i.e., lights on, impact vs. intent, full stop, teach) Considerations for creating an inclusive learning environment (e.g., helping students deal with derogatory comments from peers, supporting students who are transitioning gender)
inclusifiED: Inclusive Teaching Practices Workshop	Describe inequities in your current classroom environment and their impact on student learning Define an effective framework for inclusive design Outline inclusive learning techniques which can be applied in the classroom to help students achieve their potential	2	120 min	Reflection activity Poll Everywhere questions Small group zoom breakout discussion Large group discussion	Importance of adding structure to learning environments Using course data to determine inequities in the classroom setting Strategies to reduce inequity in the classroom setting

DEI = diversity, equity, and inclusion; STEM = science, technology, engineering, and mathematics.
